# Heat stress modulates extracellular vesicles miRNA cargo in bovine uterine fluid and uterine function

**DOI:** 10.3389/fvets.2026.1863620

**Published:** 2026-07-08

**Authors:** Salvatore Monti, Michal Andrzej Kosior, Riccardo Esposito, Valentina Palmieri, Stefania Di Giorgio, Gabriele Marino, Emanuele Capra, Anna Lange-Consiglio, Giulia Gaspari, Valentina Longobardi, Barbara Lazzari, Paola Gagni, Bianca Gasparrini

**Affiliations:** 1 Department of Veterinary Sciences (SCIVET), University of Messina, Messina, Italy; 2 Vespro APS, Messina, Italy; 3 Department of Veterinary Medicine and Animal Production (DMVPA), University of Naples Federico II, Naples, Italy; 4 Institute of Biology and Agricultural Biotechnology, National Research Council (CNR), Milan, Italy; 5 Department of Veterinary Medicine and Animal Sciences (DIVAS), University of Milan, Lodi, Italy; 6 Department of Experimental Medicine, University of Rome Tor Vergata, Rome, Italy; 7 Institute of Chemical Sciences and Technologies “Giulio Natta” (SCITEC), National Research Council (CNR), Milan, Italy

**Keywords:** dairy cattle, extracellular vesicles, fertility, heat stress, interleukin-6, microRNA, uterine fluid, uterine function

## Abstract

**Introduction:**

Heat stress (HS) impairs fertility in dairy cattle, yet the molecular mechanisms underlying uterine dysfunction remain poorly understood. This study investigated the impact of HS on the microRNA (miRNA) cargo of extracellular vesicles (EVs) derived from bovine uterine fluid (UF) and its association with reproductive performance.

**Methods:**

Environmental conditions were classified according to the temperature–humidity index (THI) recorded during the study periods: thermoneutral conditions in winter (THI: 57.7 ± 0.9; THI < 72) and heat-stress conditions in summer (THI: 76.6 ± 1.8; THI > 72). On the day of estrus (day 0), a total of 20 Holstein cows were enrolled, 10 during winter and 10 during summer. On the same day, Doppler ultrasonography was performed to assess uterine artery hemodynamics, and uterine fluid was collected for EV isolation and miRNA profiling. The conception rate was determined 30 days after artificial insemination (AI) by ultrasound. Pregnancy was defined as the visualization of an embryo with heartbeat by transrectal ultrasonography 30 days after AI.

**Results:**

Under HS, conception rates were markedly reduced. Under HS, conception rates were markedly reduced, and uterine artery resistance was increased, suggesting impaired uterine perfusion. EVs isolated from UF showed comparable size and concentration across seasons; however, their miRNA cargo was significantly altered. Small RNA sequencing identified 93 differentially expressed miRNAs, with a predominance of down-regulated miRNAs under HS. Functional enrichment analysis indicated that these miRNAs target genes involved in interleukin-6 (IL-6) signaling, lipid metabolism, and cellular communication. Notably, HS was associated with the accumulation of miRNAs potentially suppressing IL-6-related pathways and lipid oxidation. Moreover, comparison with previously published data showed that a subset of miRNAs was shared with follicular fluid EVs, suggesting coordinated regulation between ovarian and uterine compartments.

**Discussion:**

Overall, HS reshapes the molecular cargo of uterine EVs and may disrupt endometrium–embryo communication, contributing to reduced fertility. These findings provide novel insights into uterine responses to HS and highlight EV-associated miRNAs as potential biomarkers of reproductive dysfunction in dairy cattle.

## 1 Introduction

Heat stress (HS) represents an increasing concern in modern dairy production systems, arising from the combined effects of climate warming and intensive selection for milk yield. Indeed, the intensive genetic selection has led to increased metabolic rate and hence reduced thermotolerance. Heat stress occurs when animals are unable to adequately dissipate excess body heat, disrupting thermoregulatory homeostasis. This condition markedly compromises the economic sustainability of dairy cattle breeding by negatively affecting both productive performance (1) and reproductive efficiency (2, 3). HS is known to induce immunosuppression (4), predisposing animals to reproductive disorders such as mastitis, retained fetal membranes, and metritis (5–7). Moreover, HS interferes with the hypothalamic–pituitary–gonadal axis (8), ovarian cyclicity (9–11), and oocyte competence (12).

To mitigate the negative effects of HS on oocyte competence, embryo transfer (ET) has been widely adopted during the hot season in cattle (13, 14). However, the uterus is also a critical target of HS, and alterations in the uterine environment may impair embryo survival and development. Under HS conditions, embryonic development can be compromised due to modifications in the uterine milieu (15, 16) or reduced implantation capacity (17, 18), leading to increased embryonic mortality and abortion rates (19, 20). In addition, HS has been shown to affect endometrial physiology by altering the expression of genes related to heat shock proteins and antioxidant responses (15), reducing protein secretion, and increasing prostaglandin production, thereby impairing luteal function and early pregnancy maintenance (21, 22). Furthermore, HS during pregnancy can alter uterine vascularization, reducing nutrient delivery to the fetus and impairing its growth (23).

Extracellular vesicles (EVs), membrane-enclosed vesicles secreted by somatic cells, carry a wide array of bioactive molecules and play a regulatory role in reproductive function (24). EVs have been identified in several reproductive tissues and fluids, including the uterus (25–30), where they contribute to the establishment of an optimal microenvironment for sperm transport and endometrial preparation for blastocyst implantation (31). These vesicles transport proteins, lipids, and non-coding RNAs, particularly microRNAs (miRNAs), and are key mediators of embryo–maternal communication (32). *In vitro* studies have demonstrated that supplementation with uterine and oviduct-derived EVs improves blastocyst quality, modulates lipid metabolism, enhances antioxidant activity, and regulates gene expression related to DNA methylation (33, 34).

Despite growing evidence supporting the role of uterine EVs in embryo–endometrium communication (35, 36), no studies have yet directly investigated the relationship between the molecular cargo of uterine fluid-derived EVs and fertility outcomes under HS conditions. In particular, it remains unclear whether HS affects the miRNA cargo of EVs in the uterine environment and how these changes may contribute to reduced reproductive performance.

The aim of this study was to evaluate the impact of HS on the molecular profile of uterine EVs at the moment of estrus and its relationship with bovine fertility by comparing miRNA cargo, conception rates after artificial insemination (AI), and uterine artery vascularization in cows under thermoneutral or heat stress conditions. Specifically, it was hypothesized that HS alters uterine vascularization and the miRNA profile of EVs contained in the uterine fluid (UF), potentially contributing to the reduced fertility observed in dairy cows exposed to HS.

## 2 Materials and methods

### 2.1 Ethical approval

The Ethical Animal Care and Use Committee of the University of Messina (Italy) approved the experimental design and all animal procedures (PG 16/2024, December 23rd, 2024).

### 2.2 Animals management and temperature–humidity index (THI)

The present study was conducted on a commercial dairy farm located in Ragusa (Southern Italy), housing approximately 400 Holstein cows. Cows were managed under consistent husbandry conditions, milked twice daily, and fed a total mixed ration (TMR) consisting of corn silage, alfalfa, and dry grass. The diet was supplemented twice daily with soybean meal and corn concentrate to meet the nutritional requirements of lactating dairy cattle. Fresh water was provided ad libitum. A data logger (Sainlogic FT-083, Sainlogic, USA) was installed in the feeding area at the head height of the animals to continuously monitor ambient temperature and relative humidity. The THI was calculated using the following formula: THI = (1.8 × AT + 32) − [(0.55–0.0055 × RH) × (1.8 × AT + 32) − 58].

where AT is the ambient temperature expressed in degrees Celsius, the term (1.8 × AT + 32) corresponds to the conversion of temperature values to degrees Fahrenheit, and RH represents the relative humidity expressed as a fraction of unity. A THI of 72 or higher has been widely used as the critical threshold for the onset of heat stress in dairy cattle (4).

### 2.3 Herd fertility assessment

To determine the reproductive status of each cow, postpartum gynecological examinations and pregnancy diagnosis were performed. These included rectal palpation and transrectal ultrasonography using a multi-frequency linear transducer (AirScan Pro, DRAMIŃSKI S. A., Poland, 7.5 MHz). Postpartum examinations were conducted every 2 weeks starting from 21 DIM. The farm operated a reproductive management program with a voluntary waiting period of 60 DIM. Estrus detection was performed using a pedometer (Afiact, Afimilk Ltd., Israel). In the farm routine, the pedometers were placed on cows at the time of the first calving. Estrus-detection alerts were generated every 10 min. The AI is performed twice a day by the farmer (after the milking routine). Pregnancy diagnosis was carried out 30 days after AI; pregnancy was defined as the visualization of an embryo with a heartbeat. Conception rates (CR) at the first and second AI were recorded.

### 2.4 Experimental design and animal enrollment

The impact of HS was evaluated at both the herd level, in terms of fertility, and at the uterine level, by analyzing EV distribution and cargo in UF collected during winter (W) and summer (S). According to similar peer-reviewed studies, a sample size of 10 animals per season was chosen to minimize the number of subjects used, thereby prioritizing animal welfare. The total number of animals selected for the fertility evaluation represents the maximum available on the farm that met the inclusion criteria during the experimental period.

Herd fertility was assessed by monitoring CR following AI during W (*n* = 119) and S (*n* = 117), corresponding to periods characterized by low (<72) and high (>72) THI, respectively.

Twenty multiparous lactating Holstein cows, clinically healthy (without signs of mastitis, lameness, systemic diseases or reproductive tract disorders), aged between 3 and 5 years, and ranging from 40 to 70 DIM, were included in the study. The animals were equally divided into two groups (*n* = 10) based on season (winter and summer). The W group included animals in the postpartum period between February and March 2025. The S group included animals from August to September 2025. For this study, cows were enrolled following an OvSynch protocol. The OvSynch protocol involved an injection of GnRH (lecirelin, Dalmarelin, Fatro S.p.a) on day 0, followed by an injection of PGF2α (d-cloprostenol, Estrotek, A. T. I S.r.l) after 7 days, and a second injection of GnRH 48 h later. On day 10 of the protocol, 24 h after the last GnRH administration and at the time of AI, animals showing estrus were selected after gynecological examination confirming pedometer-detected heat (Afiact, Afimilk Ltd., Israel). Blood samples were collected to evaluate P4 levels using a point-of-care device (Speed Progesterone – Speed Reader, Virbac Diagnostics, France) to confirm low P4 levels consistent with estrus.

These animals underwent uterine artery flowmetry to evaluate seasonal differences in vascular parameters. Subsequently, UF was collected for EV isolation and characterization of their miRNA cargo, as described below.

### 2.5 Uterine fluid collection, ultrasound flow evaluation

Uterine lavage was performed at estrus under caudal epidural anesthesia using 2% procaine (Procamidor, Izo Srl, Italy). The procedure involved the infusion of 40 mL sterile 0.9% sodium chloride solution into the uterine body using a 60 mL syringe attached to a disposable plastic infusion rod (Insemination Catheter, KRUUSE, Denmark). To minimize contamination from vaginal discharge, the infusion rod was covered with a sanitary plastic sleeve during insertion. The recovered UF was collected in sterile tubes for further processing.

**Figure 1:**
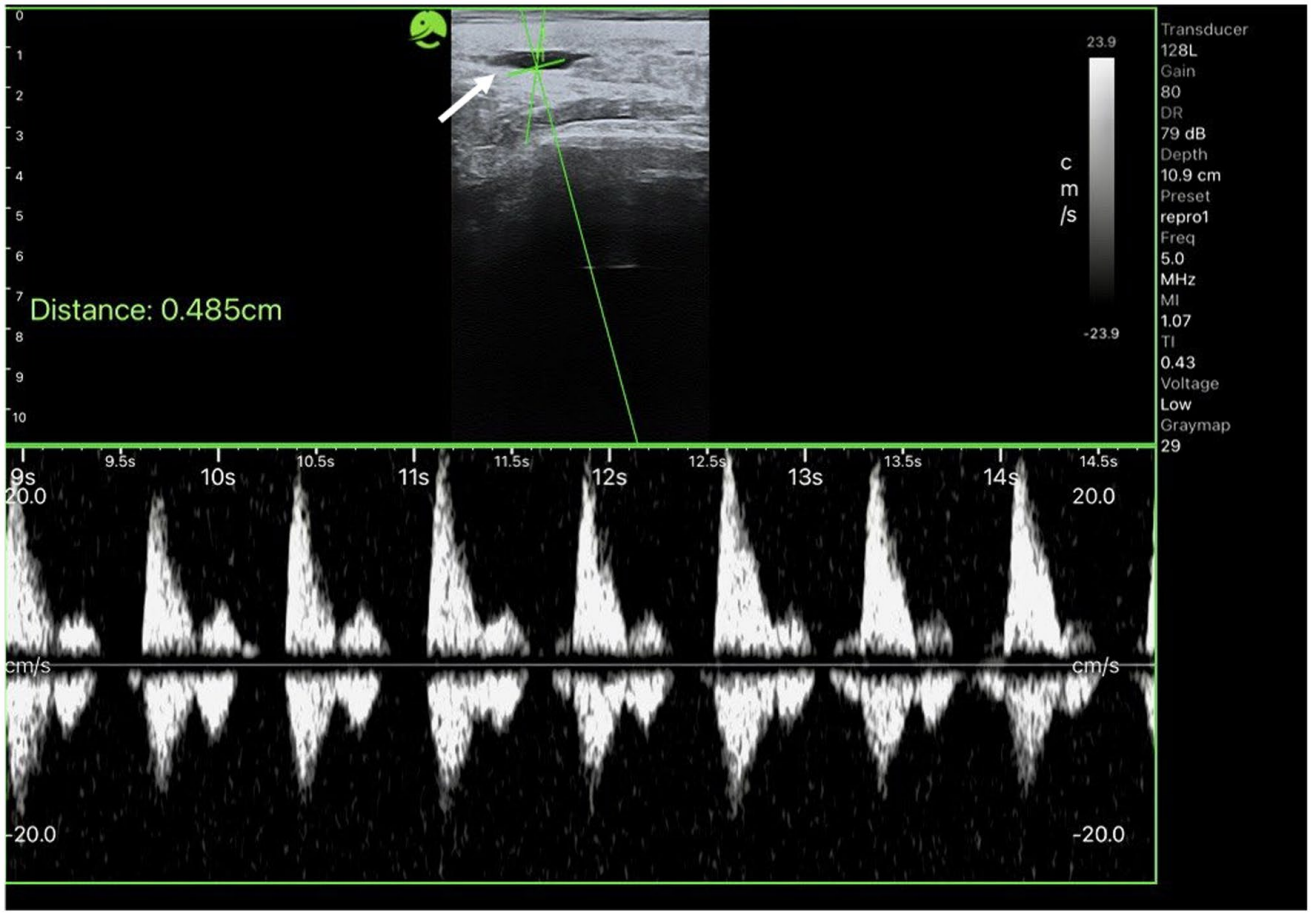
Ultrasonographic visualization of the uterine artery (with arrow) and Doppler flow profile.

Following fluid collection, uterine artery blood flow was evaluated on the same side as the preovulatory follicle. For Doppler examination, performed by linear transducer (AirScan Pro, DRAMIŃSKI S. A., Poland, 7.5 MHz), the aorta was scanned in its caudal region to identify the internal iliac artery, from which the uterine artery originates (37). The Doppler gate was adjusted to match the vessel diameter, and a high-pass filter was set at 100 Hz to eliminate signals from slowly moving tissues and vessel wall motion. Blood flow velocity waveforms were obtained at an interrogation angle between 20° and 60°. Resistance index (RI), vessel diameter, and time-averaged maximum velocity (TAVmax) were recorded. A representative image of the ultrasonographic evaluation is shown in Figure 1.

### 2.6 Extracellular vesicles isolation from uterine fluid

Extracellular vesicles (EVs) were isolated from UF samples (*n* = 10 per season) by sequential differential centrifugation. Samples were first centrifuged at 300×g for 10 min at 4 °C to remove dead cells and large debris, followed by centrifugation at 6,000×g for 20 min at 4 °C to eliminate larger vesicles and residual debris in a on-farm mobile laboratory. After centrifugation, the tubes were stored at 4 °C and sent to the laboratory.

A final ultracentrifugation step was performed at 100,000×g for 1 h at 4 °C using an OptimaX ultracentrifuge (Beckman Coulter, Milan, Italy) to pellet EVs. The resulting pellets were resuspended in 200 μL phosphate-buffered saline (PBS) and stored at −20 °C until EV characterization and RNA extraction.

### 2.7 Nanoparticle tracking analysis

The size distribution and concentration of UF-derived EVs were assessed by Nanoparticle tracking analysis (NTA) using a NanoSight NS300 system (Malvern Technologies, Malvern, UK) equipped with a 532 nm laser. Samples were diluted in filtered PBS (1:200, v/v) to achieve an optimal particle concentration (20–100 particles/frame). For each sample, three videos of 60 s were recorded and analyzed using NTA software (version 3.4). Mean, mode, and median particle sizes were obtained from each video and used to calculate EV concentration, expressed as nanoparticles/mL.

### 2.8 Dot blot analysis

Ten UF-derived samples from W and ten from S were pooled separately. Aliquots (0.8 μL) of pooled EVs were spotted onto nitrocellulose membranes (PROTAN BA 85, 0.45 μm; Whatman, Germany) and allowed to dry at room temperature (RT) for 15 min. Non-specific binding sites were blocked with 4% bovine serum albumin (BSA) in TBS-T (0.05% Tween-20).

Membranes were incubated for 2 h at RT under orbital shaking with mouse IgG primary antibodies diluted 1:500 in TBS-T containing 2% BSA: anti-ALIX (clone C11, Santa Cruz Biotechnology, USA), anti-TSG101 (clone C-2, Santa Cruz Biotechnology, USA), anti-CD63 (clone H5C6, BD Pharmingen, USA), anti-CD81 (clone B-11, Santa Cruz Biotechnology, USA), and anti-Calnexin (clone TO-5, Sigma-Aldrich, Italy).

After three washes (5 min each) with TBS-T, membranes were incubated with horseradish peroxidase-conjugated anti-mouse secondary antibody (Jackson ImmunoResearch, UK) diluted 1:2500 in TBS-T containing 2% BSA for 1 h. Following three additional washes (5 min each), signals were detected using Clarity Western ECL Substrate (Bio-Rad, Italy) and visualized with a Chemidoc XRS imaging system (Bio-Rad, Italy). The characterization assays performed for the vesicles are compliant with the MISEV2023 guidelines (38).

### 2.9 Transmission electron microscopy

As previously reported (39), the isolated EVs were fixed for 1 h at room temperature (RT) in 2% paraformaldehyde and 2.5% glutaraldehyde in 0.1 M sodium cacodylate buffer (pH 7.4). Samples were then post-fixed with 1% OsO_4_ and 1.5% potassium ferrocyanide in 0.1 M cacodylate buffer for 1 h on ice under dark conditions. Samples were washed, stained with 0.5% uranyl acetate, and dehydrated through a graded ethanol series. Specimens were infiltrated for 2 h in a 1:1 (v/v) ethanol–resin (Araldite–Epon) mixture, followed by two incubations in 100% Epon resin for 1 h each. Polymerization was performed at 60 °C for 48 h. Ultrathin sections (70 nm) were collected on 300-mesh uncoated copper grids using an Ultracut E microtome (Reichert, Austria). Sections were examined with a Zeiss LEO 912ab energy-filtering transmission electron microscope operating at 120 kV. Digital images were acquired using a CCD-BM/1 K camera system with iTEM software (Olympus Soft Imaging Solutions).

### 2.10 RNA extraction

RNA extraction was performed from isolated EVs (*n* = 10 per season). EVs were lysed in Trizol (Invitrogen, Carlsbad, CA, USA), followed by the addition of chloroform and centrifugation according to the manufacturer’s protocol.

The aqueous phase containing RNA was further purified using the NucleoSpin miRNA kit (Macherey–Nagel, Germany), without additional DNase digestion step and directly used for library preparation with the QIAseq miRNA Library Kit RNA quality and concentration were assessed using an Agilent 2,100 Bioanalyzer (Agilent Technologies, Santa Clara, CA, USA). Purified RNA samples were stored at −80 °C until further analyses.

### 2.11 Library preparation and sequencing

Small RNA libraries were generated using the QIAseq miRNA Library Kit (QIAGEN, Venlo, Netherlands), following the manufacturer’s instructions, using QIAseq miRNA 96 Index IL adapters and 14 PCR amplification cycles. The library preparation workflow selectively enriches small RNAs, and no evidence of genomic DNA interference was observed in library quality controls or downstream sequencing analyses. Libraries were purified using a Pippin Prep instrument (Sage Science, MA, USA). Library quality and concentration were evaluated using an Agilent 2,100 Bioanalyzer (Agilent Technologies, Santa Clara, CA, USA). Sequencing was performed on an Illumina NovaSeq X platform using 150-cycle paired-end reads. Sequencing data are available in the Sequence Read Archive (SRA) under BioProject accession number PRJNA1453246.

### 2.12 Data analysis

Raw sequencing data were quality-checked using FastQC^1^ and trimmed with Trimmomatic (version 0.32). Processed reads were analyzed using miRDeep2 (version 2.0.0.5) for miRNA identification, using *Bos taurus* miRNA sequences to support known miRNA detection. miRNA expression was quantified using the miRDeep2 quantifier module, while differential expression analysis between winter (W) and summer (S) groups was performed using the EdgeR Bioconductor package (version 3.6) (40). Differentially expressed miRNAs (DE-miRNAs) were defined according to statistical significance using a False Discovery Rate (FDR) threshold < 0.05 only, and no additional log fold change threshold was applied. To identify shared mechanisms between compartments, the 93 DE-miRNAs identified in the present experiment were intersected with the 83 DE-miRNAs previously reported by our research group (41). Predicted target genes for both the seasonal DE-miRNAs (W vs. S) in UF and the resulting overlapping miRNAs between UF and FF were identified using miRWalk2.0 (42), using homologous bovine miRNAs as input identifiers. Target genes were then analyzed using Cytoscape (version 3.2.1) with the ClueGO plugin (version 2.3.5) for Gene Ontology (GO) enrichment analysis (43).

Conception rates for the first and second AI were analyzed separately. For each AI order, seasonal differences (winter vs. summer) were assessed using the chi-square test. First and second AI were not included in the same statistical model; thus, no repeated-measures structure was applied. Statistical significance was set at *p* < 0.05. Seasonal differences in uterine blood flow parameters, including RI, vessel diameter, and time-averaged maximum velocity (TAVmax), were evaluated using two-sided independent-samples *t*-tests. Equality of variances between groups was assessed using Levene’s test, and Welch’s correction was applied when the assumption of equal variances was not met. As three comparisons were performed, statistical significance was determined using a Bonferroni-adjusted threshold (*p* < 0.0167).

## 3 Results

During winter (February–March 2025), THI ranged from 54 to 59, with a mean value of 57.7 ± 0.9 and never exceeded 72. Cows evaluated during this period had a mean DIM of 57.3 ± 11.1, a mean parity of 3.1 ± 1.0, and a mean daily milk production of 45.4 ± 7.0 kg/day. During summer (August–September 2025), THI ranged from 74 to 89, with a mean value of 76.6 ± 1.8, with values above 72 for 60 days of the monitoring period, confirming exposure to heat stress conditions. Cows evaluated during this period had a mean DIM of 61.2 ± 8.3, a mean parity of 3.1 ± 1.0, and a mean daily milk production of 41.3 ± 7.1 kg/day. CR was evaluated in a total of 118 AI events during winter and 115 AI events during summer. During winter, 51 events corresponded to first AI and 29 to the second AI. During the summer, 42 events corresponded to the first AI and 29 to the second AI. Specifically, conception rate at first AI decreased from 39.2% in winter to 16.7% in summer, whereas conception rate at second AI decreased from 55.2 to 17.2%. Significant differences were observed between seasons for THI and conception rate at both first and second AI (*p* < 0.001). Uterine fluid was collected from a synchronized subset of 10 cows per season.

### 3.1 Uterine artery flowmetry

Selected animals presented typical signs of estrus, including clear, stringy vaginal mucus, increased uterine tone, and a preovulatory follicle (1.6–1.8 cm in diameter), in the absence of a corpus luteum and abnormal uterine contents. All cows included in the UF collection subset had P4 concentrations reported as <1 ng/mL on the day of estrus, confirming luteolysis and estrus. The point-of-care device used for progesterone assessment does not provide exact numerical values below this threshold but reports concentrations as <1 ng/mL.

Uterine blood flow parameters differed between seasons. Resistance index (RI) was significantly higher in summer compared to winter (0.8 ± 0.1 vs. 0.6 ± 0.1, *p* = 0.00041), indicating increased vascular resistance. Similarly, time-averaged maximum velocity (TAVmax) was higher in summer (22.0 ± 1.11 cm/s) than in winter (18.4 ± 3.2 cm/s, *p* = 0.0065). Vessel diameter was lower in summer (0.3 ± 0.1 cm) compared to winter (0.4 ± 0.1 cm), although this difference was not significant after Bonferroni correction (Table 1).

**Table 1:** Seasonal variations in uterine blood flow parameters.

Variable	Summer Mean ± s.d.	Winter Mean ± s.d.
RI	0.80 ± 0.10^a^	0.60 ± 0.10^b^
Diameter (cm)	0.30 ± 0.10^a^	0.40 ± 0.10^b^
TAVmax (cm/s)	22.0 ± 1.11^a^	18.4 ± 3.22^a^

Mean ± standard deviation of resistance index (RI), vessel diameter, and time-averaged maximum velocity (TAVmax) measured during summer and winter. Different superscript letters (a, b) denote statistically significant differences between seasons.

### 3.2 Extracellular vesicles characterization

**Figure 2:**
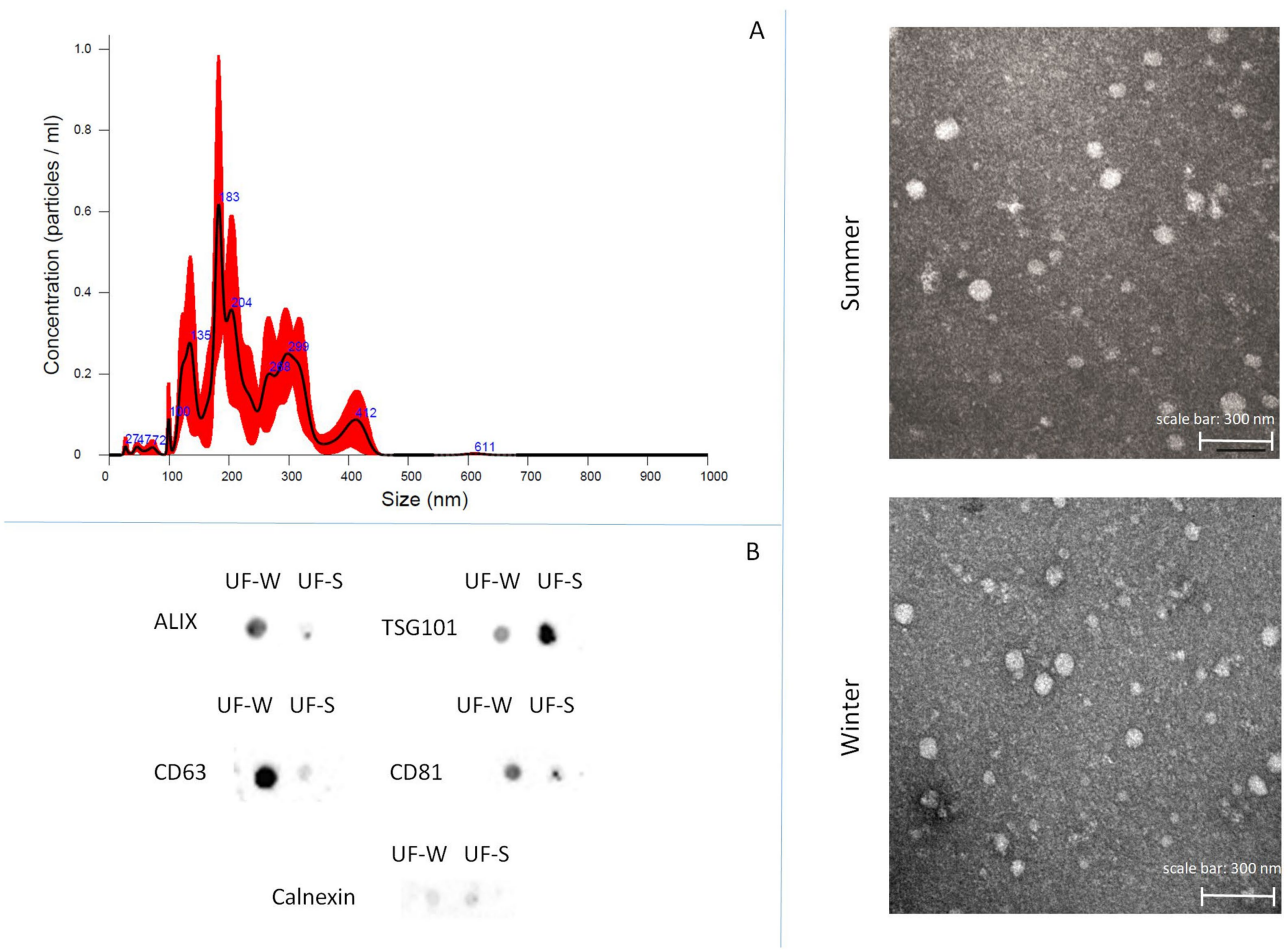
EVs isolated from uterine fluid (UF) collected during winter (W) and summer (S) season by: **(A)** Nanoparticle Tracking Analysis (NTA), **(B)** Dot Blot for EVs internal markers (TSG101 and Alix), membrane markers (CD63 and CD81); and Calnexin as marker of cell contamination in EVs preparation. **(C)** Transmission Electron Microscopy that revealed typical morphologies characteristic of vesicle (scale bar: 300 nm).

Nanoparticle tracking analysis revealed a comparable size distribution (mean diameter of 244.2 nm, 235.1 nm for winter and 253.2 nm for summer) and particle concentration (4.2 × 10^10^ particles/mL, 5.2 × 10^10^ particles/mL for winter, 5.2 × 10^10^ particles/mL for summer) of UF-derived EVs obtained from winter and summer samples (Supplementary file S1), with a representative NTA profile shown in Figure 2A. EV identity was further supported by dot blot analysis, used as a complementary characterization method. Due to the limited amount of material recovered from individual samples, Western blot analysis was not feasible; therefore, pooled preparations were used for dot blot analysis. This approach allowed the detection of EV markers while minimizing sample consumption and reducing variability among individual replicates. All antibodies used in this study had been previously validated for specificity in independent reports (44, 45), ensuring selective recognition of target proteins. Dot blot analysis confirmed the presence of canonical EV markers (CD63, CD81, ALIX, and TSG101), while showing negligible signal for the endoplasmic reticulum marker Calnexin. The image was intentionally overexposed to facilitate visualization of the sample spots despite the absence of a detectable Calnexin signal, indicating the absence of significant cellular contamination (Figure 2B).

Finally, transmission electron microscopy confirmed the presence of vesicular structures with morphology consistent with EVs (Figure 2C).

### 3.3 Small RNA sequencing

**Figure 3:**
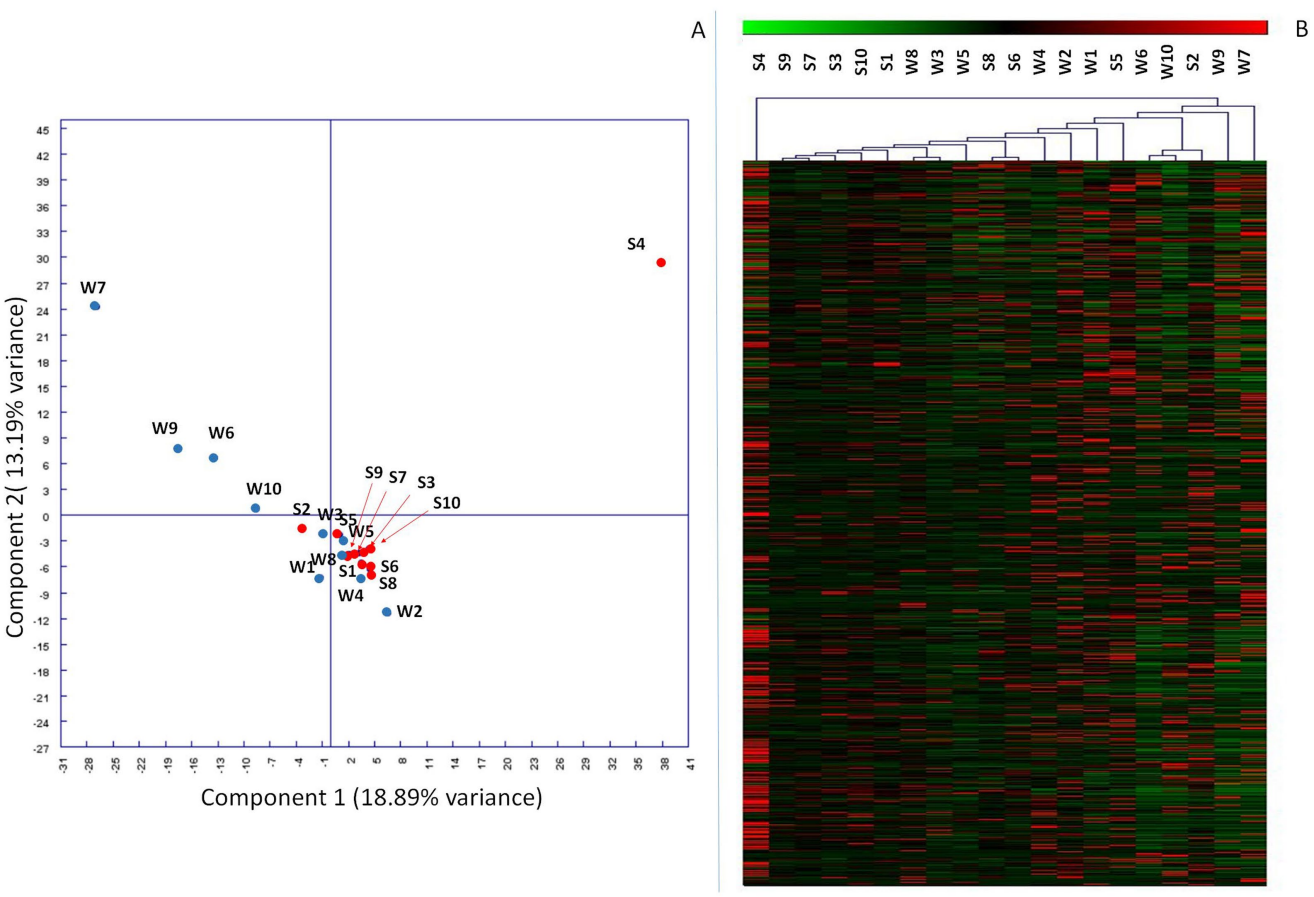
**(A)** Principal Component Analysis and **(B)** hierarchical clustering of samples (extracellular vesicle from uterine fluid UF-EVs) analyzed in the hot summer (S) and cold winter (W) seasons based on their miRNA cargo.

The EVs isolated from uterine fluid samples collected in winter and summer (*n* = 10 per group) were analyzed for their miRNA content. An average of approximately 47.4 million reads were obtained for each sample (45.0 for winter and 49.9 for summer), of which about 21.0% were assigned to miRNAs (see Supplementary file S2 for sequencing statistics). In total, 808 *Bos taurus* miRNAs were identified (with at least one count in three samples). Considering the relative expression of these miRNAs, Principal Component Analysis (PCA) and hierarchical clustering analysis showed only a partial separation between samples in relation to season (Figures 3A,B).

Most of the winter samples clustered separately in the PC1 (explaining about 19% of the variance) and were largely distributed, whereas the miRNA cargo of UF-EVs in S was more homogeneous, except for the S4 sample. The UF contained EVs showed a specific miRNA cargo with 93 differentially expressed miRNAs (DE-miRNAs - False Discovery Rate FDR < 0.05) between summer and winter (Supplementary file S3). UF-derived EVs from animals bred in S had 17 over-expressed and 76 under-expressed miRNAs compared to winter. The top five most significant (based on False Discovery Rate FDR) over- and under-expressed miRNAs are reported in Table 2.

**Table 2:** The top five most significantly over- and under-expressed miRNAs (based on False Discovery Rate FDR) between summer (S) and winter (W) seasons.

miRNA	logFC	logCPM	FDR
bta-miR-12034	−5.2122	10.85173	8.42E−06
bta-miR-2898	−6.65509	8.606473	1.21E−05
bta-miR-2358	−8.30395	6.585665	1.48E−05
bta-miR-2305	−6.18422	6.05126	2.55E−05
bta-miR-2478	−4.99477	8.387212	4.79E−05
bta-miR-148b	1.670723	10.96857	0.003634
bta-miR-2285 t	3.070351	6.021239	0.008483
bta-miR-2285i	2.763791	3.196685	0.01121
bta-miR-2284w	2.90351	4.175256	0.01121
bta-miR-10166-5p	2.77129	1.53574	0.01602

In the table the logarithmic Fold Change (logFC), the logarithmic Count Per Million reads (logCPM) and the False Discovery Rate (FDR) are reported.

**Figure 4:**
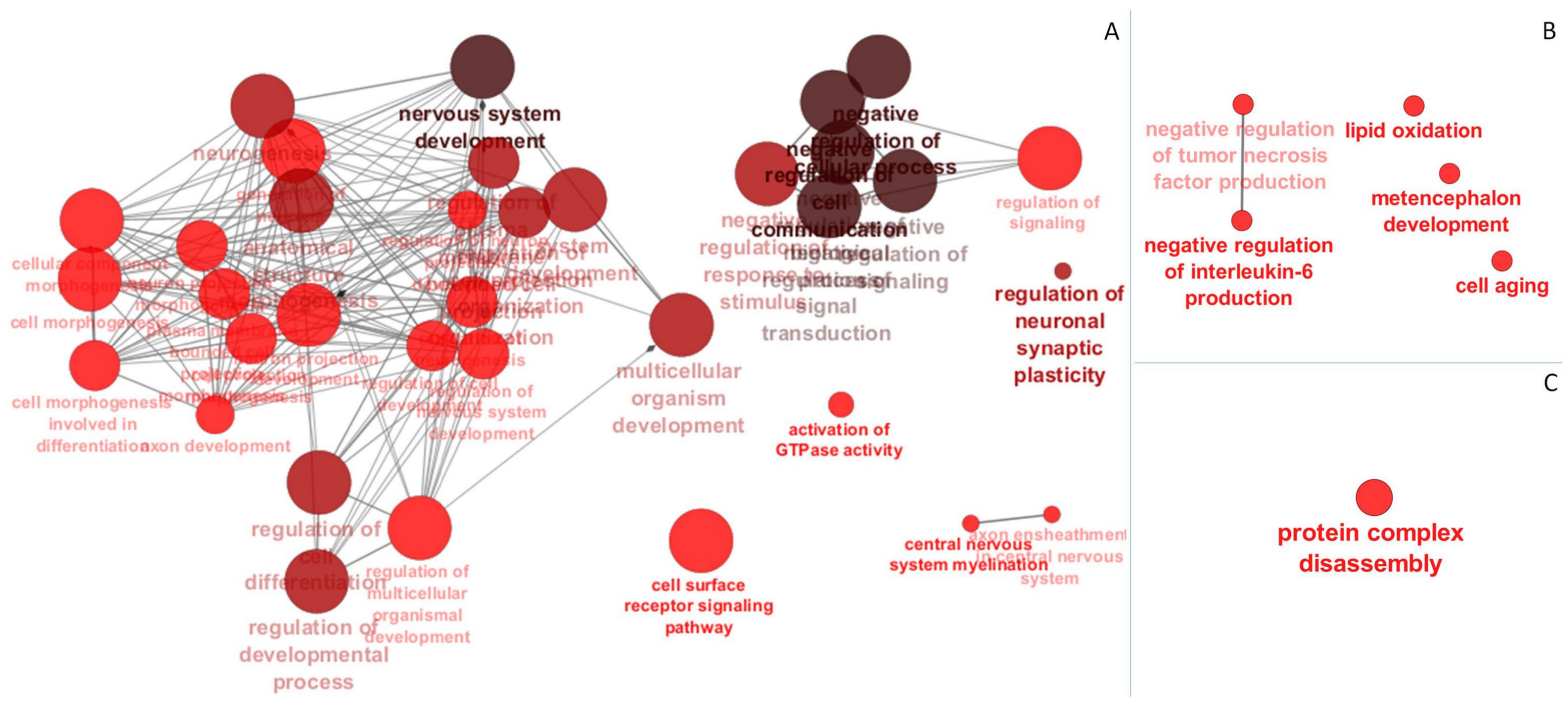
Gene Ontology (GO) Analysis on target genes (top 10 for each miRNA) for: **(A)** total DE-miRNA (*n* = 93) **(B)** over-expressed and **(C)** under-expressed miRNAs in summer season (S) from extracellular vesicles isolated from uterine fluid (UF-EVs).

Gene Ontology (GO) analysis was performed on DE-miRNA target genes (Supplementary file S4). As in silico analysis targets hundreds of genes for each DEmiRNA, GO analysis was performed considering the first 10 target genes for each DE-miRNA (sorted by 3’UTR binding energy and longest consecutive pairing). Across the 93 DE-miRNAs identified between seasons, the predicted target genes are involved in the regulation of cellular processes as well as in cell communication, morphogenesis, and differentiation (Figure 4A). The HS induces accumulation of miRNAs (over-expressed miRNA in S, *n* = 17) targeting genes related to interleukin-6 (IL-6) production, aging, and lipid oxidation (Figure 4B), and depletion of miRNAs (under-expressed miRNA in S, *n* = 76) targeting genes involved in protein complex disassembly (Figure 4C), Supplementary file S5.

**Figure 5:**
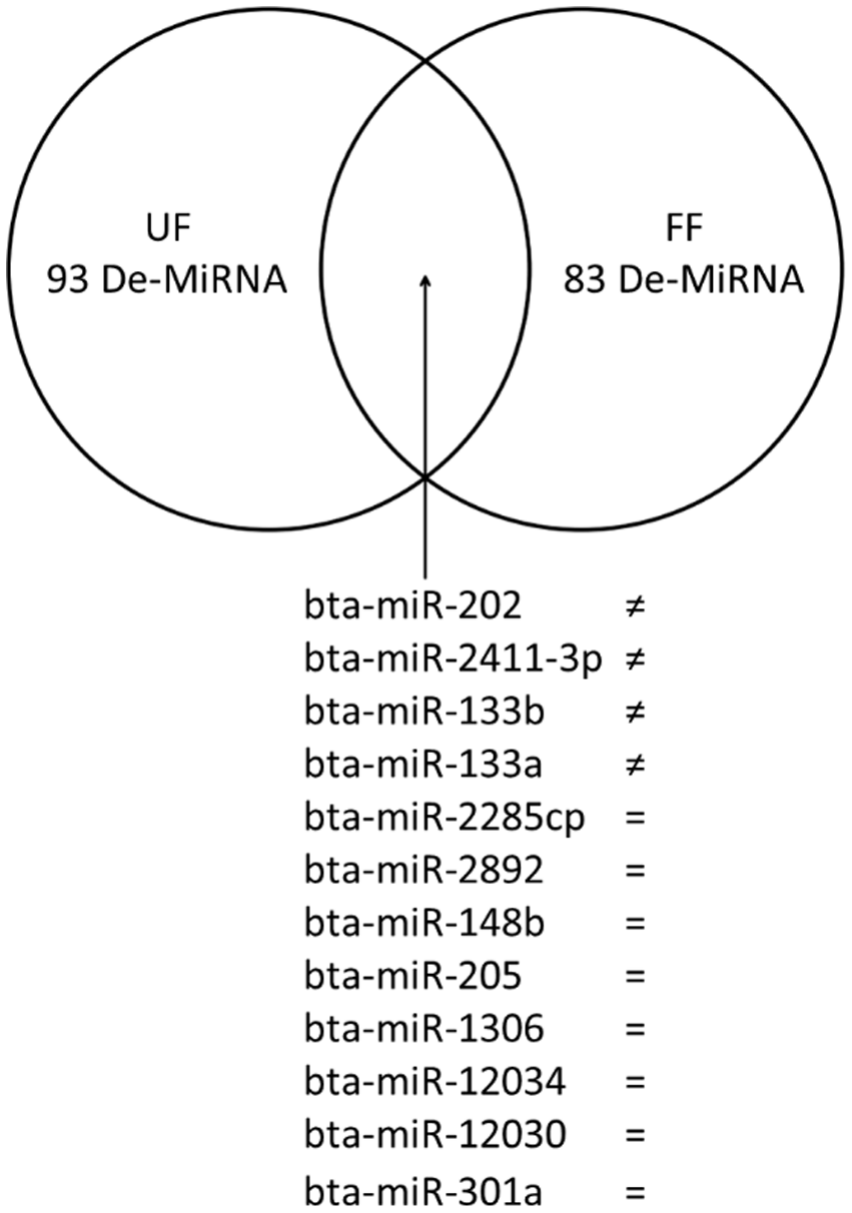
Venn diagram illustrating the overlap of miRNAs with seasonal variations between Uterine fluid (UF) and Follicular Fluid (FF). Intersection identifies 12 shared miRNAs. The symbol “=” indicates a concordant trend, while “≠“a discordant trend.

**Figure 6:**
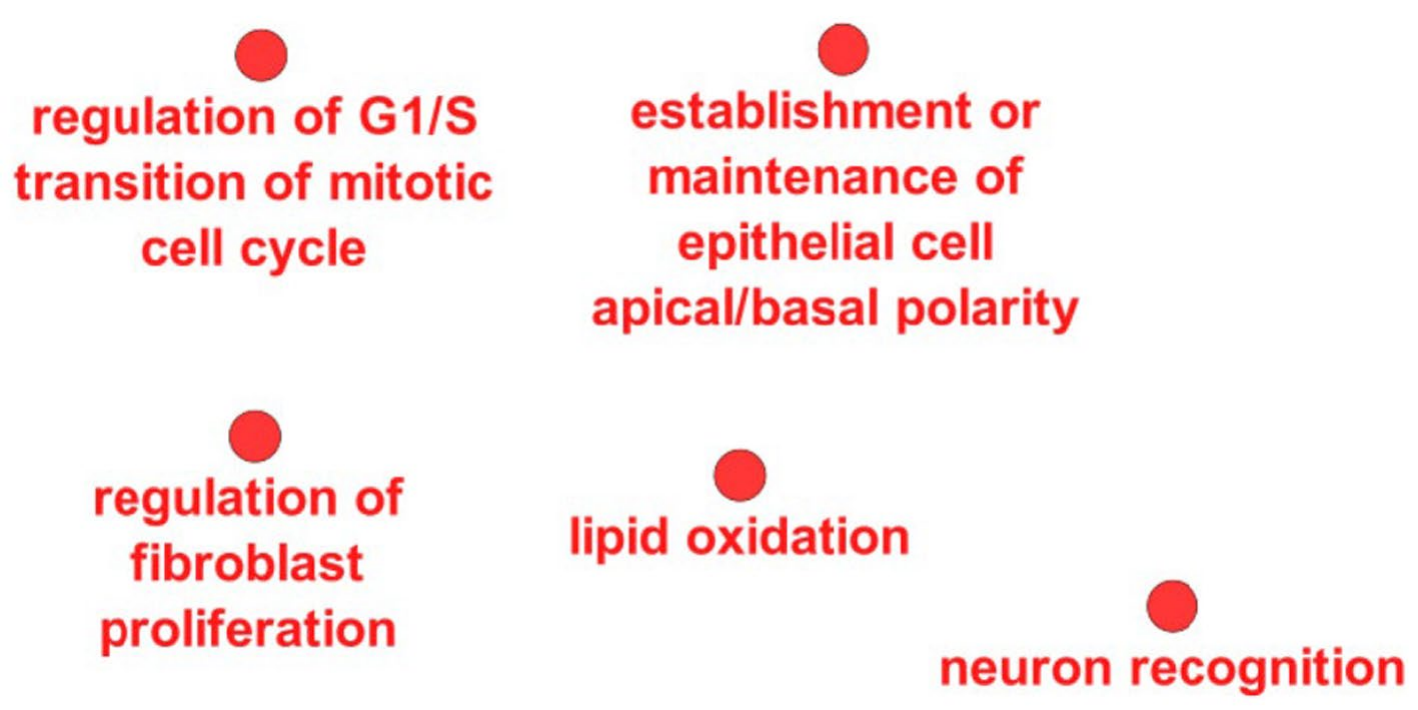
Gene Ontology (GO) Analysis on target genes regulated by the miRNAs overlapping between those with heat stress induced variations identified in extracellular vesicles isolated from Uterine fluid (UF) and Follicular Fluid (FF).

The intersection between the DE-miRNAs identified in the present study and the previously published follicular fluid dataset (41) revealed an overlap of 12 miRNAs exhibiting seasonal variations was observed between the two datasets. Most of these miRNAs showed concordant seasonal trends (8/12), whereas 4 out of 12 displayed discordant trends, as shown in Figure 5. The predicted target genes of the 12 miRNAs overlapping between the FF and UF were calculated from the miRWalk Database. The first 20 target genes (sorted by 3’UTR binding energy and longest consecutive pairing), for each FF and UF shared DE-miRNA, were taken. The resulting gene list was subjected to Gene Ontology (GO) enrichment analysis. The analysis yielded 5 significantly enriched biological processes (Figure 6).

## 4 Discussion

The present study aimed to investigate the impact of HS on the miRNA cargo of EVs in bovine uterine fluid and its relationship with reproductive performance. The results demonstrate that HS is associated with a marked reduction in fertility, alterations in uterine vascularization, and significant changes in the molecular cargo of uterine EVs.

In the current study, the mean THI during summer was 76.6 ± 1.8, exceeding the threshold of 72 all monitored days, which is considered the critical limit for thermal comfort in dairy cattle (46), confirming that cows were exposed to HS. In agreement with previous studies (2, 38, 41), HS was associated with a substantial decrease in conception rates. Indeed, conception rate decreased by more than 20 percentage points during summer at both first and second AI, indicating a biologically relevant impairment of reproductive efficiency. This reduction is likely the result of multiple and interacting mechanisms, including altered endocrine function, impaired estrus expression, reduced oocyte and embryo competence, increased oxidative stress, and compromised uterine receptivity. In addition, uterine artery blood flow was altered during HS, as indicated by increased RI values, suggesting reduced uterine perfusion. This vascular alteration may be partly explained by the thermoregulatory response to HS. Under high environmental temperatures, peripheral vasodilation and increased blood flow to the skin and respiratory tract are required to dissipate excess body heat. This redistribution of blood flow may occur at the expense of visceral and reproductive tissues, including the uterus, thereby increasing uterine vascular resistance and reducing uterine perfusion. Such impaired vascularization may contribute to a suboptimal uterine environment by limiting oxygen and nutrient delivery to the endometrium and by altering local metabolic and inflammatory responses. These changes may negatively affect sperm transport, uterine receptivity, early embryo development, and embryo survival (47). Therefore, reduced uterine perfusion may represent one of the physiological mechanisms contributing to the marked decrease in conception rate observed under HS conditions. Furthermore, although the relationship between uterine vascularization and local EVs is still unelucidated, it is possible to hypothesize that altered blood flow may modulate the EV content of UF. To our knowledge, no studies have yet directly examined the link between uterine vascularization and EV profiles in bovine uterine fluid. However, changes in uterine blood flow are likely to shape the endometrial microenvironment by modulating the supply of oxygen, nutrients, hormones, and immune signals. In HS conditions, reduced perfusion may therefore impair uterine receptivity while simultaneously altering EV release, cargo composition, and intercellular communication. Although still unproven, this mechanism offers a strong rationale for targeted investigation and a promising direction for future research (Table 3).

**Table 3:** GO terms identified for the target genes regulated by the miRNAs overlapping between those with heat stress induced variations identified in extracellular vesicles isolated from uterine fluid (UF) and follicular fluid (FF).

GO:	Associated genes found	Term	*p* value
8,038	AMIGO1, CNTN2, EFNB3	Neuron recognition	1.1E−02
48,145	DDR2, DHX9, FBRS, MORC3	Regulation of fibroblast proliferation	2.6E−02
34,440	ACADM, CROT, PPARA	Lipid oxidation	3.0E−02
45,197	CRB2, PTK7, VANGL2	Establishment or maintenance of epithelial cell apical/basal polarity	4.4E−02
2,000,045	ANKRD17, GIGYF2, JADE1, PSME3	Regulation of G1/S transition of mitotic cell cycle	4.6E−02

Indicated are Gene Ontology IDs (GO), associated genes found, gene ontology terms (Term), and *p* values.

Although EV size and concentration were comparable between seasons, their miRNA cargo was significantly altered under HS conditions. The mean size of EVs in our study was consistent with that previously reported in the uterine fluid of the same species (48). The PCA showed only a partial separation between the two groups. However, the observation that most of the samples collected during winter did not cluster tightly together suggests a greater individual variability under thermoneutral conditions compared to the hot season. In contrast, samples collected from animals subjected to HS clustered more closely, suggesting that HS may elicit a common and consistent response across endometrial cells.

The EVs isolated from UF in the two seasons showed a distinct miRNA cargo, with 93 differentially expressed miRNAs (DE-miRNAs) between summer and winter, of which 17 were over-expressed and 76 were under-expressed in summer. It is worth noting that bta-miR-2898 and bta-miR-2478, which were under-expressed during summer in this study, are involved in the regulation of reproductive function in various species, including cattle (49–52). In particular, within the ovary the bta-miR-2898 was found up-regulated in the early stages of corpus luteum formation, highlighting its importance in luteal development (50). Furthermore, bta-miR-2898 and bta-miR-2478 miRNAs have been identified in the plasma of pregnant cattle, suggesting a potential role with embryonic and fetal development (53). These miRNAs have also been reported to show differential expression in response to HS in bovine and buffalo (54–56). In a previous study, thermal stress was shown to increase circulating levels of miR-2898, and its expression was correlated with that of heat shock proteins (HSPs) in cattle (55). Sengar et al. (53) reported differences in the blood levels of miR-2898 and miR-2478 in Frieswal crossbred dairy cattle subjected to HS. Finally, under-expression of miR-2478 was observed in the blood of buffalo heifers exposed to HS and was strongly correlated with the expression of HSP-related mRNAs (56). Taken together, the variations of these miRNAs strongly indicate their involvement in the cellular response to HS, potentially modulating thermotolerance mechanisms.

Moreover, Gene Ontology analysis revealed that HS induces the accumulation of miRNAs targeting genes involved in interleukin-6 (IL-6) production, aging, and lipid oxidation. The over-expression of miRNAs associated with the negative regulation of IL-6 secretion in animals subjected to HS is particularly interesting, given the role of this cytokine in reproductive function in cattle (56, 57). Consistent with our findings, differential expression of miRNAs related to IL-6 pathways has also been reported in EVs from buffalo follicular fluid and associated with seasonal subfertility (58).

Interleukin-6 is produced by both the endometrium and the conceptus (59, 60) and plays a role in early embryo development, implantation, and uterine immune regulation (61, 62). However, its effects are tightly regulated, as both insufficient and excessive IL-6 levels have been associated with impaired reproductive function (63). Taken together, these observations suggest that alterations in miRNAs targeting IL-6-related pathways in uterine EVs under HS conditions may contribute to dysregulation of the uterine environment and reduced fertility. However, further studies are needed to directly assess IL-6 dynamics in this context.

Another pathway identified by GO analysis as potentially regulated by DE-miRNAs was lipid oxidation. Given the inhibitory role of miRNAs on their mRNA targets (64, 65), the increased expression of miRNAs targeting genes involved in lipid oxidation pathways during summer may suggest a metabolic shift toward reduced lipid catabolism. Alterations in lipid metabolism may be linked to mitochondrial oxidative stress and to compromised prostaglandin synthesis and energetic homeostasis (58–60, 66).

In addition, IL-6 signaling and lipid metabolism are known to influence endothelial function and angiogenesis (67, 68) and these pathways may represent concomitant factors that, together with the increased RI observed in the uterine artery during summer, contribute to a condition of reduced uterine perfusion.

The list of DE-miRNAs identified in this study was compared with miRNAs isolated from EVs derived from preovulatory follicular fluid under HS conditions in a previous study (38, 41), revealing 12 DE-miRNAs shared between the two compartments. Among these, the majority displayed concordant seasonal trends, suggesting a synchronized and conserved response to HS across the two compartments. Conversely, the discordant expression observed for four miRNAs highlights the physiological and cellular divergence between the two microenvironments. These opposite trends could be attributed to compartment-specific regulatory requirements reflecting how a single miRNA may exert different functional roles depending on the specific cellular target. GO analysis of the miRNAs shared between FF and UF showed that the target genes potentially regulate biological processes closely linked to reproduction, including lipid oxidation, establishment or maintenance of epithelial cell apical/basal polarity, and regulation of the G1/S transition of the mitotic cell cycle.

The identification of shared miRNAs between separate but functionally connected compartments, such as the ovarian follicle and the endometrium, reinforces the concept that coordinated regulatory mechanisms exist between the ovary and uterus, which are necessary for proper reproductive function. Among the pathways regulated by these shared miRNAs, the G1/S transition of the mitotic cell cycle is particularly relevant, as this process is essential for granulosa cell proliferation and early embryonic cell division (69–71). Furthermore, this finding is consistent with the altered progression of the G1 phase and the reduction in the S-phase cell population observed in granulosa cells cultured under HS conditions and subjected to CAT knockdown (72).

Moreover, the observed variations in the regulation of genes involved in lipid metabolism, known to play a key role in reproductive function, may indicate a general metabolic reorganization associated with HS.

Overall, the identification of altered miRNAs potentially involved in the regulation of genes associated with these pathways supports the hypothesis that miRNAs may jointly regulate critical functions related to fertility. In addition, the negative impact of HS on fertility is likely to have a multifactorial etiology, with similar pathways operating across different biological compartments.

## 5 Conclusion

In conclusion, to the best of our knowledge, this study provides the first evidence of HS-induced alterations in the miRNA cargo of EVs isolated from UF in dairy cattle, which are associated with reduced fertility and alterations in uterine artery vascularization. Among the differentially expressed miRNAs (DE-miRNAs), 17 were over-expressed and 76 were under-expressed during summer (S). Some of these DE-miRNAs have been previously associated with the response to HS in cattle and buffalo.

Furthermore, our findings indicate that HS leads to the accumulation of miRNAs targeting genes involved in IL-6 production, aging, and lipid oxidation, and to the depletion of miRNAs targeting genes involved in protein complex disassembly. These alterations suggest that HS may affect uterine secretory and metabolic functions, impair endometrium–embryo communication, and ultimately reduce fertility.

Understanding the molecular alterations induced by HS in the uterine environment may contribute to the development of strategies aimed at mitigating its negative effects on reproductive performance in dairy cattle.

## Data Availability

The sequencing data for this study can be found in the Sequence Read Archive (SRA) under BioProject accession number PRJNA1453246 (https://www.ncbi.nlm.nih.gov/bioproject/PRJNA1453246).

## Footnotes

1
http://www.bioinformatics.babraham.ac.uk/projects/fastqc/

## Supplementary material

SUPPLEMENTARY FILE S1. Nanoparticle tracking analysis (size distribution (nm) and particle concentration (particles/mL)) of UF-derived EVs obtained from winter (W) and summer (S) samples.

SUPPLEMENTARY FILE S2. Statistic of small-RNA sequencing of extracellular vesicle isolated from Uterine Liquid (UL) collected in the Winter (W) and Summer (S) seasons.

SUPPLEMENTARY FILE S3. Differentially expressed miRNAs DE-miRNAs between UF-derived EVs obtained from winter (W) and summer (S) samples.

SUPPLEMENTARY FILE S4. In silico analysis of DE-miRNA target genes using miRWalk2.0 software.

SUPPLEMENTARY FILE S5. Node tables of Gene Ontology GO analysis of predicted miRNAs target genes.
