# Supplementary material for: Heat stress modulates extracellular vesicles miRNA cargo in bovine uterine fluid and uterine function
Source: Front Vet Sci. 2026 Jul 8;13:1863620. doi: 10.3389/fvets.2026.1863620 (PMC13388032; doi:10.3389/fvets.2026.1863620)
Supplement: SUPPLEMENTARY FILE S2. — Statistic of small-RNA sequencing of extracellular vesicle isolated from Uterine Liquid (UL) collected in the Winter (W) and Summer (S) seasons. [file Table_2.DOCX]

**Supplementary File S2.** Statistic of small-RNA sequencing of extracellular vesicle isolated from Uterine Liquid (UL) collected in the Winter (W) and Summer (S) seasons.

| **Sample** | **Total reads** | **QC-passed reads** | **miRNA reads** |
| --- | --- | --- | --- |
| UL-W1 | 18688143 | 18146349 | 1352314 |
| UL-W2 | 49508096 | 45933376 | 2954327 |
| UL-W3 | 42108229 | 40047905 | 5825029 |
| UL-W4 | 69892264 | 66692195 | 1694170 |
| UL-W5 | 76350694 | 75386187 | 18399295 |
| UL-W6 | 29577037 | 28702593 | 613675 |
| UL-W7 | 36216685 | 35181771 | 149298 |
| UL-W8 | 68080651 | 64241602 | 5473773 |
| UL-W9 | 22364832 | 21652588 | 199577 |
| UL-W10 | 37150879 | 35963171 | 1027149 |
| UL-S1 | 42338598 | 40583186 | 8029135 |
| UL-S2 | 46592217 | 44856190 | 1609927 |
| UL-S3 | 66654665 | 65280207 | 26645422 |
| UL-S4 | 77449214 | 74972429 | 45737785 |
| UL-S5 | 54320561 | 52848502 | 18913999 |
| UL-S6 | 33083943 | 31858684 | 8158982 |
| UL-S7 | 46061009 | 43989914 | 16515635 |
| UL-S8 | 30915705 | 29859168 | 19576784 |
| UL-S9 | 40271695 | 38077859 | 10590698 |
| UL-S10 | 61060000 | 59170520 | 5865474 |
| Average | 4.74E+07 | 4.57E+07 | 9.97E+06 |
